# Risk of Axillary Nerve Injury in Standard Anterolateral Approach of Shoulder: Cadaveric Study

**DOI:** 10.5704/MOJ.1811.001

**Published:** 2018-11

**Authors:** W Kongcharoensombat, P Wattananon

**Affiliations:** Department of Orthopaedics, Lerdsin General Hospital, Bangkok, Thailand

**Keywords:** axillary nerve, anterolateral shoulder approach, axillary nerve injury

## Abstract

**Introduction:** The anterolateral acromion approach of the shoulder is popular for minimally invasive plate osteosynthesis (MIPO) technique. However, there are literatures describing the specific risks of injury of the axillary nerve using this approach. Nevertheless, most of the studies were done with Caucasian cadavers. So, the purpose of this study was to evaluate the risk of iatrogenic axillary nerve injury from using the anterolateral shoulder approach and further investigate the location of the axillary nerve, associated with its location and arm length in the Asian population that have shorter arm length compared to the Caucasian population.

**Materials and Methods:** Seventy-nine shoulders in fourty-two embalmed cadavers were evaluated. The bony landmarks were drawn, and a vertical straight incision was made 5cm from tip of the acromion (anterolateral approach), to the bone. The iatrogenic nerve injury status and the distance between the anterolateral edge of the acromion to the axillary nerve was measured and recorded.

**Results:** In ten of the seventy-nine shoulders, the axillary nerve were iatrogenically injured. The average anterior distance was 6.4cm and the average arm length was 30.2cm. The anterior distance and arm length ratio was 0.2.

**Conclusion:** Our results demonstrated that the recommended safe zone at 5cm from tip of acromion was not suitable with Asian population due to shorter arm length, compared to Caucasian population. The location of axillary nerve could be predicted by 20% of the total arm-length.

## Introduction

The anterolateral acromion approach of the shoulder is a popular approach for the treatment of proximal humeral fractures, especially in minimally invasive plate osteosynthesis (MIPO) technique. The advantage of this approach is that it allows direct access to the lateral fracture planes for proximal humeral fracture reduction, plate placement and screw fixation^1-12^ with potentially greater preservation of soft tissue structures than the deltopectoral approach^13^.

However, there are reports in the literature describing the specific risks of the axillary nerve injury in this approach^1-5^, as, in the anatomical course of the axillary nerve, it wraps around the surgical neck of the humerus. Many studies have investigated and reported that it lies an average of 5cm distal to the acromion. Nevertheless, most of the studies had been done with Caucasian cadavers with one of the studies that reported about the significant correlation between arm length and position of axillary nerve^4^.

So, the purpose of this study was to evaluate the risk of iatrogenic axillary nerve injury from using the anterolateral shoulder approach and to further investigate the location of the axillary nerve, in relation to its anatomical location and arm length in the Asian population.

## Materials and Methods

Fourty-two embalmed cadavers were used in the study. Both shoulders were dissected in all, but 79 shoulders were included (five shoulders were too decayed and anatomy could not been identified). There was no previous history of trauma or surgery around the shoulder in the specimens.

**Fig. 1:**
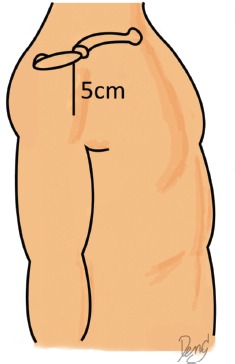
Cadaver in supine position. Bony landmarks were marked. Incision was made for 5cm from tip of acromion.

**Fig. 2:**
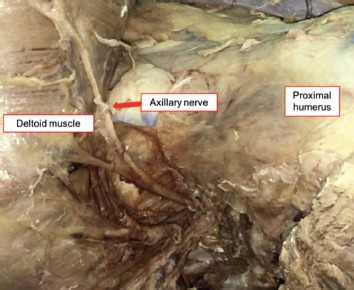
Deltoid muscle reflected and the Axillary nerve identified.

With the cadavers in the supine position, the position of the acromion, clavicle and proximal humerus were marked. The distance between the anterolateral edge of the acromion to the lateral humeral condyle was measured and recorded as the arm length. The 5cm incision was made as a vertical straight line from the anterolateral edge of the acromion (anterolateral approach), deep to the bone, with the end of incision marked by silk (Fig. 1). Dissection was then performed and the deltoid muscle released from the acromion. The inner surface of the deltoid muscle was exposed, and the sub-deltoid fascia was excised to expose the axillary nerve (Fig. 2).

Pins were inserted through the axillary nerve to outline its course on the outer surface of the deltoid muscle. Then, the deltoid muscle was sutured back to its original anatomic position. The iatrogenic nerve injury was identified (presence of sharp cut on the axillary nerve that occurred from the anterolateral approach incision) and the distance between the anterolateral edge of the acromion to the axillary nerve was measured and recorded. All the measurements were made using the Digital Vernier Caliper.

The data were recorded using Microsoft Excel 2016 version. Data were shown as means ± SD. Statistical analysis was done using Graph Pad Prism version 7.02 software. Analysis between arm length and distance of axillary nerve from tip of acromion was performed using a Pearson's correlation coefficient. Values of p<0.05 were considered significant.

## Results

The mean age of all the persons (classified as cadavers in the study) at the time of death was 76 year (range: 21-99 years), and 54.8% were male. Most of them were Thai and immigrants from China (Table I). The axillary nerves were found to have been iatrogenically injured in 10 of 79 shoulders (12%). The means length between the axillary nerve and anterolateral edge of the acromion was 6.39±0.99cm (range: 4.6 to 8.2cm). The average arm length was 30.17±0.03cm (range: 26.1 to 34.5cm). There was a significant correlation between the arm length and the length between the axillary nerve and anterolateral edge of acromion, with an average index at 0.2 (95% CI: 0.205-0.217) (Table II).

**Table I:** Demographic data

Variables	All patient (n=42)
Age (mean)	76 (21-99)
Gender	
Male	23 (54.8%)
Female	19 (45.2%)
Ethnicity	Thai and Migrant Chinese

**Table II:** Axillary nerve injury

Code	Sex	Age	Side	Nerve Injury Yes/No	Nerve location (cm)	Total arm length (cm)	Nerve location / total arm length
1	F	77	R	No	7.1	29.3	0.2
2	F	77	L	No	7.3	29.6	0.2
3	M	76	L	No	7.5	30.2	0.2
4	M	76	R	No	8.2	30.1	0.3
5	M	92	L	No	6.5	30.2	0.2
6	M	92	R	No	8.1	30	0.3
7	F	50	R	No	4.9	29.2	0.2
8	F	50	L	No	6.9	29.6	0.2
9	M	85	R	No	7.5	34.1	0.2
10	M	85	L	No	7	33.2	0.2
11	F	88	R	No	6.6	29.1	0.2
12	F	88	L	No	7.5	29.7	0.3
13	M	76	R	No	6.6	29.2	0.2
14	M	76	L	Yes	5	29.1	0.2
15	F	82	L	Yes	4.9	26.6	0.2
16	F	82	R	No	5.9	27.2	0.2
17	M	84	L	No	6.5	30.1	0.2
18	M	84	R	No	6.5	29.8	0.2
19	M	99	L	No	6.7	31.2	0.2
20	M	99	R	No	7.1	31.5	0.2
21	F	89	R	Yes	4.6	27.2	0.2
22	F	89	L	Yes	4.9	28.2	0.2
23	F	94	R	No	5.5	28.2	0.2
24	F	94	L	Yes	4.6	26.8	0.2
25	F	79	R	No	5.3	26.5	0.2
26	F	79	L	Yes	4.5	26	0.2
27	M	62	R	No	7.2	32.2	0.2
28	M	62	L	No	7	32	0.2
29	F	91	R	No	6	27	0.2
30	F	91	L	No	5.5	26.5	0.2
31	F	73	L	No	7	29.2	0.2
32	F	73	R	No	5.2	28.5	0.2
33	M	81	L	No	8.1	34.2	0.2
34	M	81	R	No	7.3	34.5	0.2
35	M	97	L	No	7.5	32	0.2
36	M	97	R	No	6.4	31.5	0.2
37	M	71	L	No	8.2	33.2	0.2
38	M	71	R	No	7.7	32.5	0.2
39	F	89	L	No	5.5	29.3	0.2
40	F	89	R	Yes	4.8	27.4	0.2
41	M	77	L	No	6.5	31.1	0.2
42	M	77	R	No	7.1	31.4	0.2
43	M	71	R	No	5.5	30.8	0.2
44	M	71	L	No	5.7	31.2	0.2
45	F	63	R	Yes	4.9	30.2	0.2
46	F	63	L	No	5.4	30.5	0.2
47	M	64	R	No	7	29.5	0.2
48	M	64	L	No	7.1	30.3	0.2
49	M	86	L	No	6.3	32.2	0.2
50	M	86	R	No	7.2	31.8	0.2
51	F	90	R	Yes	4.6	29.5	0.2
52	F	90	L	No	5.8	30.4	0.2
53	M	66	L	No	6.7	31.5	0.2
54	M	66	R	No	7.2	31	0.2
55	F	78	R	No	5.9	29.5	0.2
56	M	92	L	No	6	30.2	0.2
57	M	92	R	No	6.5	31	0.2
58	F	68	L	No	4.9	28	0.2
59	F	68	R	No	6.1	28.5	0.2
60	F	73	L	No	5.5	28.2	0.2
61	F	73	R	No	6.4	28	0.2
62	M	84	L	No	6.5	31	0.2
63	M	84	R	No	6.6	30.5	0.2
64	M	71	L	No	6.8	31.5	0.2
65	M	71	R	No	7.4	31	0.2
66	F	75	R	No	6.2	28.5	0.2
67	F	75	L	No	6.5	28.8	0.2
68	M	77	L	No	7.1	34.2	0.2
69	M	77	R	No	8.1	33.5	0.2
70	F	68	R	No	6.2	29.5	0.2
71	M	66	R	No	7.6	29.5	0.3
72	M	66	L	No	7.1	30.2	0.2
73	M	83	L	No	7.2	31.8	0.2
74	M	83	R	No	7.2	32	0.2
75	F	21	L	No	5.6	30.5	0.2
76	M	76	L	No	6.1	33.2	0.2
77	M	76	R	No	6.1	33.4	0.2
78	M	54	R	No	5.6	28.5	0.2
79	M	54	L	No	5.6	29	0.2
	M = 23 F = 19 Total = 42	77.3 (n=79)		10/69	6.4	30.2	0.2

## Discussion

The anatomy of the axillary nerve has been described by many authors. The distance from acromion varied from 3 to 9cm, depending on the reference point of acromion and shoulder positions. There are some muscles which have a supplementary function in elevation of the shoulder, such as rotator cuff muscle, pectoralis major muscle, etc. However, the anterior branch of axillary nerve is still important to preserve function of the shoulder. The anterior branch of the axillary nerve provides the motor function of deltoid muscle. The incidence of axillary nerve injury in deltoid splitting approach is about 7% but this in anterolateral approach is yet to be reported^1-7^.

With reference to the anterolateral approach of the shoulder, many textbooks state that the safe zone of axillary nerve is 5cm from tip of the acromion. However, cadaveric studies show that the distance of acromion to axillary nerve varies between from 4 to 8cm. So, the 5-cm-guideline does not guarantee absolute protection^8^. In this study, we found that the nerve injury was 10 of 79 shoulders (12%).

In terms of the location of axillary nerve, Cetik *et al* found that the average distance from anterior acromion was 6.1cm and the ratio between distance from anterior acromion and lateral epicondyle was 0.2. This is verified by the present study that the average was 6.3cm and the ratio was 0.24. However, we found that in 10 of 79 shoulders, the nerve locations were less than 5cm. From this fact, 5cm from the tip of acromion was to be considered not safe in all cases but the location of the nerve could be more safely estimated from 20% of arm length.

This study demonstrated that the anterolateral surgical approach should be performed meticulously to identify and protect the axillary nerve. However, the axillary nerve might be at risk all the time, not only when stab incision is made but also in reduction of fracture or plate insertion. Gardner *et al* described the location of the nerve relative to the PHILOS plate. They found that the axillary nerve usually lay on the calcar screw hole^8^. This information should help the surgeon to be aware of the risk when reducing, drilling and inserting the calcar screw.

Several limitations exist in the current study. First, this is cadaveric study, so it may not reflect the same findings in patients. Second, though, we used the Digital Vernier Caliper for accuracy of measurement, human error could still occur. Third, it may not be possible to avoid the risk in patients who have displaced or comminuted fracture of proximal humerus resulting in injury-related deformity.

## Conclusion

Our results demonstrated that the recommended safe zone at 5cm from the tip of acromion in anterolateral surgical approach of the shoulder was not suitable in the average Asian population due to shorter arm length, compared to the Caucasian population. The location of the axillary nerve could be more accurately be predicted by 20% of the total arm length.

## Conflict of Interest

The authors declare no conflicts of interest.
